# A Coaching-Based Training for Underrepresented Mentors in STEM

**DOI:** 10.3390/educsci15030289

**Published:** 2025-02-26

**Authors:** Molly E. Tuck, Kaylee A. Palomino, Julie A. Bradley, Margaret Mohr-Schroeder, Luke H. Bradley

**Affiliations:** 1 Department of Transformative Learning, University of Kentucky, Lexington, KY 40506, USA; 2 Office of Academic Affairs and Innovation, Kansas State University, Manhattan, KS 66506, USA; 3 Department of Neuroscience, University of Kentucky, Lexington, KY 40536-0298, USA

**Keywords:** near-peer mentoring, coaching, mentor training, underrepresented minorities, high school, undergraduate

## Abstract

As an approach, coaching-based models have been demonstrated to enhance student self-efficacy, improve grades, and increase retention and graduation rates. Coaching-based training models are also key in mentor development, focusing on open-ended questions and active listening to create supportive environments where mentees can independently find solutions. This approach not only builds mentors’ communication and leadership skills but also enhances their adaptability and problem-solving abilities. For underrepresented groups in STEM, such training positions mentors as knowledge facilitators, helping bridge gaps in mentorship experiences and bolstering confidence in their roles, thereby contributing to a more inclusive and effective learning ecosystem. This study investigates the impact of a coaching-based approach to near-peer mentor training within the UK START program, focusing on high school student participants. Interviews revealed significant benefits, including enhanced communication skills, particularly in asking open-ended questions and avoiding judgmental language. Mentors also reported improved composure in stressful situations, often utilizing techniques such as deep breathing to manage emotions during interactions with young campers. Additionally, participants experienced personal growth, seeing themselves as leaders and role models, which they attributed to the mentorship training. The role affirmed their confidence in their STEM knowledge and sparked interest in future mentorship roles. These findings suggest that structured coaching-based training can build a supportive environment, benefiting both mentors and mentees.

## 1. Introduction

Authentic learning experiences have long been recognized to increase motivation to learn, obtain and synthesize a more comprehensive understanding of content and its application, increase knowledge of professionalism, and experience working through actual challenges (Edmonds-Cady & Sosulski, 2012; Mohr-Schroeder et al., 2014; Roberts et al., 2018; L. H. Bradley et al., 2021). Learning environments which affirm and encourage students’ identities, self-efficacy, and agency are critical in promoting positive STEM identities and success in learning and retaining STEM content (Singer et al., 2020; Jackson et al., 2021; Ford et al., 2023). In addition, negative and limited authentic learning experiences lead to a loss of students’ sense of belonging, eventually increasing their overall disinterest in STEM (Rainey et al., 2018; Vela et al., 2020; Jackson et al., 2021). Thus, introducing students early, and throughout their education, to role models is vital in developing a stronger sense of belonging, especially from minoritized populations in STEM fields (Atkins et al., 2020; Maiorca et al., 2021).

Mentorship in STEM is a structured relationship where a more experienced individual (mentor) provides direction, support, and expertise to an individual with less experience (mentee) in these disciplines (Crisp & Cruz, 2009). As Lunsford et al. (2017) simply put, “mentoring relationships are embedded in the educational process in higher education” (p. 316). From mentorship between students and their peers to those between students and their professors to even veteran faculty members mentoring new professors, systems within higher education embrace the benefits of forming and preserving these important professional relationships. An especially popular form of mentorship across colleges and universities is near-peer mentorship, in which a senior student provides support to other learners on similar levels of education (Anderson et al., 2015; Akinla et al., 2018; Anderson et al., 2019). This partnership can be advantageous to mentees who benefit from shared wisdom and experience but also to mentors who are positioned as having “valuable perspectives and expertise to contribute, which empowers them to engage in their own learning and development” (Chan & Luo, 2022, p. 875). For these reasons, opportunities aimed at providing access for underrepresented populations in higher education, such as the Gains in the Education of Math and Science program (Anderson et al., 2015), often include some form of near-peer mentorship.

However, while there is widespread recognition of the importance of mentorship in personal and professional development among higher education students, there is a need for more studies that evaluate the effectiveness of these programs, including high-quality peer-to-peer mentorship training in higher education (Morales et al., 2020). This is especially true for training that utilizes a coaching approach (McFarlane, 2023). Therefore, programs in higher education that utilize near-peer mentorship must intentionally seek best practices for preparing students to guide and coach their peers. One such program is the University of Kentucky’s (UK) STEM Through Authentic Research and Training (START) Program, which aims to strengthen and diversify the STEM community by enriching research and innovation related to STEM education at postsecondary institutions. A component of the UK START Program is a week of service, where student participants (called START Apprentices) in the summer cohort act as mentors to elementary-aged campers working on STEM-based tasks. Prior to working as camp mentors, the START Apprentices cohort receives a 3 h training on foundational coaching skills to apply to their near-peer mentor roles. This study examines the START Apprentices’ perceptions of the peer training and the impact of working with students in a near-peer mentoring role.

## 2. Benefits of Mentorship

### 2.1. Mentee Benefits

Research has proven peer and near-peer mentorship to be extremely effective for mentees, both in academic as well as social outcomes. For example, Lapon and Buddington (2023) found that mentees’ perceptions of relatedness, competence, and belonging improved significantly after participating in a peer mentorship program. Students receiving mentorship from peers are also more likely to be retained at higher rates (Foy & Keane, 2018; Hall et al., 2020; Lapon & Buddington, 2023). There are also mental wellness benefits, as studies on near-peer mentoring have shown reduction in mentees’ stress levels (Akinla et al., 2018), likely due to the built-in support systems of these programs.

Significant research has also examined the outcomes of peer mentoring programs specifically designed for underrepresented populations. Peer and near-peer mentorship can be an effective strategy for increasing belongingness among marginalized populations in specific majors (Pon-Barry et al., 2017) as well as institutional engagement offerings, like undergraduate research (Luedke et al., 2023). Luedke et al.’s (2023) examination of Project Scholar, a first-year experience focused on introducing Students of Color and former participants of programs like Upward Bound to undergraduate research, found that mentors shared valuable navigational capital for first-generation students and that participants benefited from having mentors who looked like their mentees. Likewise, Kalpazidou Schmidt and Faber (2016) highlighted how a mentoring program in STEM may help female students gain understanding of gender structure issues in highly competitive academic environments.

### 2.2. Mentor Benefits

However, mentors benefit from these relationships as well with many mentors experiencing growth and maturation in relation to their new leadership role. Tenenbaum et al. (2014) studied the experiences and outcomes of near-peer mentors from the Walter Reed Army Institute of Research and Gains in the Education of Math and Science program, which has trained 240 near-peer mentors over 11 years. The authors found the near-peer mentors had learned “the skills necessary for professional success. These skills included adaptability to change, professional attire, timeliness, being responsible, workplace performance, and content knowledge” (Tenenbaum et al., 2014, p. 380). Similarly, Kalpazidou Schmidt and Faber (2016) explored how mentors benefit from their roles, noting improved leadership qualities, the development of collegiality, improved mentoring and communication skills, as well as the opportunity to capitalize on personal strengths.

Additionally, mentors often appreciate having the opportunity to uplift their peers (Colvin & Ashman, 2010) and enjoy seeing the transition in their mentees (Foy & Keane, 2018). Another benefit of peer and near-peer mentoring, as opposed to faculty or staff to student mentoring, is that both individuals are in a place of growth and development that can promote reciprocal learning opportunities. In fact, mentors noted that their roles even led them to become better students themselves (Colvin & Ashman, 2010). Finally, Anderson et al. (2019) noted the long-term career orientations of individuals involved in such programs, finding that approximately half of former near-peer mentors from the Walter Reed Army Institute of Research and Gains in the Education of Math and Science program pursued STEM-careers and the other half returned to STEM-based academic programs.

In summary, near-peer mentoring offers significant benefits to mentors, including professional growth, enhanced leadership skills, and improved communication abilities. These relationships foster a unique environment where both mentors and mentees can learn and develop together, creating a reciprocal learning experience. Mentors not only gain valuable professional skills but also find personal satisfaction in supporting their peers and witnessing their growth. This dual benefit underscores the importance of near-peer mentoring programs, which will be further explored in the following section, focusing on the specific near-peer mentoring program that is the focus of this study.

## 3. UK START Program

**Figure 1.**
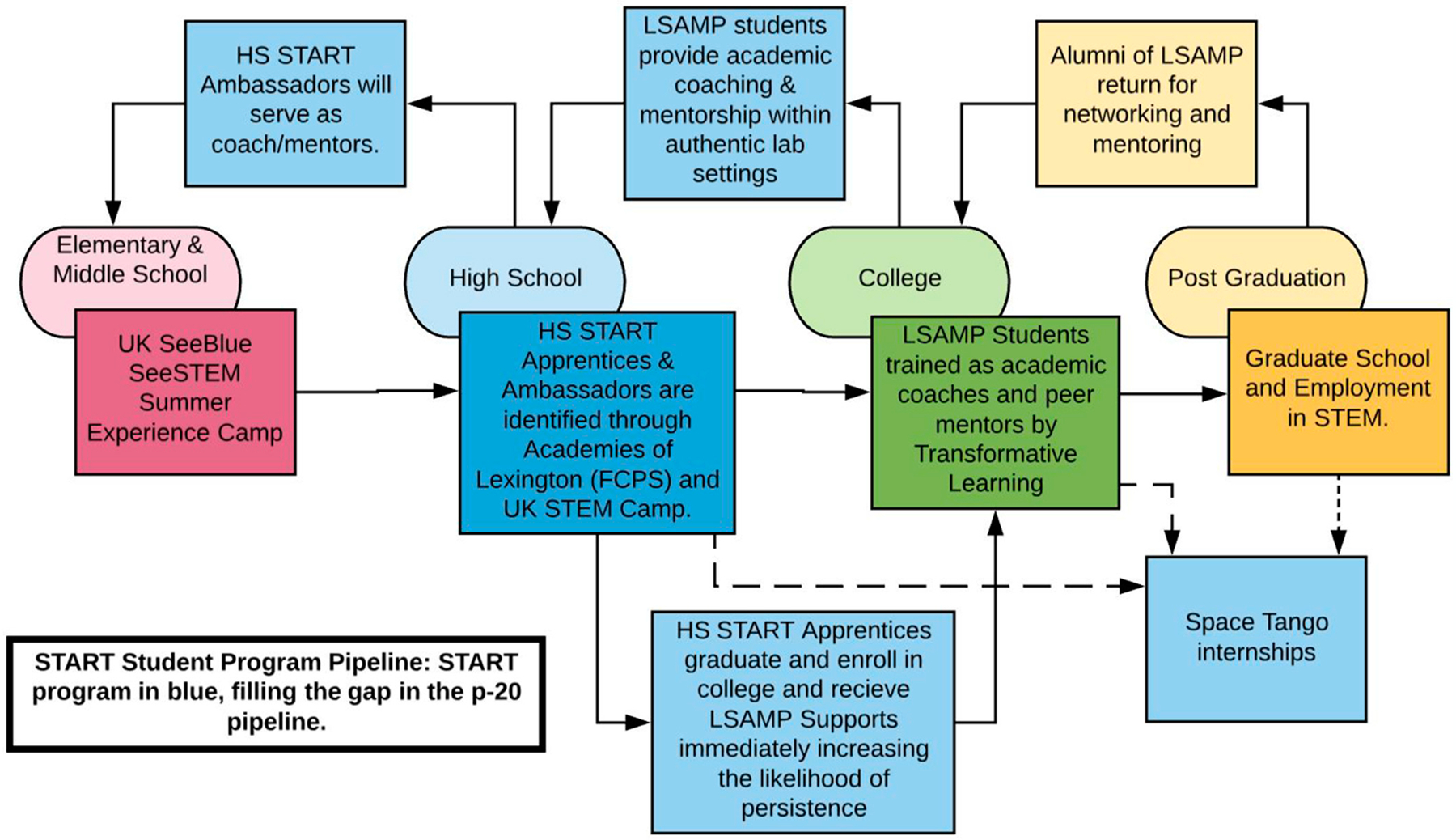
START student program pipeline (adapted from L. H. Bradley et al., 2021).

The UK START Program is a campus-wide and community partnership program seeking (see Figure 1) to enhance the recruitment and retention of first-generation and underrepresented students in STEM by implementing a comprehensive, P-12 outreach initiative. This program is designed to engage high school students (START Apprentices, grades 11–12) by bringing them to the UK campus, where they gain authentic, year-round laboratory learning experiences and access to campus supports (L. H. Bradley et al., 2021). The START Program incorporates near-peer mentoring from undergraduate student research mentors, often members of the Louis Stokes Alliance for Minority Participation (LSAMP) program at the University of Kentucky, creating a clear path for START Apprentices to see themselves as future undergraduates and scientists, while also offering mentoring and leadership opportunities for undergraduate students (L. H. Bradley et al., 2021; J. A. Bradley et al., 2024). To continue the p20 pipeline, the high school START Apprentices serve as near-peer mentors to the See Blue See STEM Summer Camp for elementary and middle school students (L. H. Bradley et al., 2021). The use of near-peer mentoring is critically important for the program’s sustainability because it helps build a STEM identity and culture, thereby becoming a pathway for underrepresented students. Finally, START Apprentices also gain access to campus support services, such as integrated success group coaching and professional development workshops hosted by coaching staff from the University of Kentucky’s Department of Transformative Learning under the Office of the Vice President for Student Success (L. H. Bradley et al., 2021).

### 3.1. UK START Peer Mentor Training

**Figure 2.**
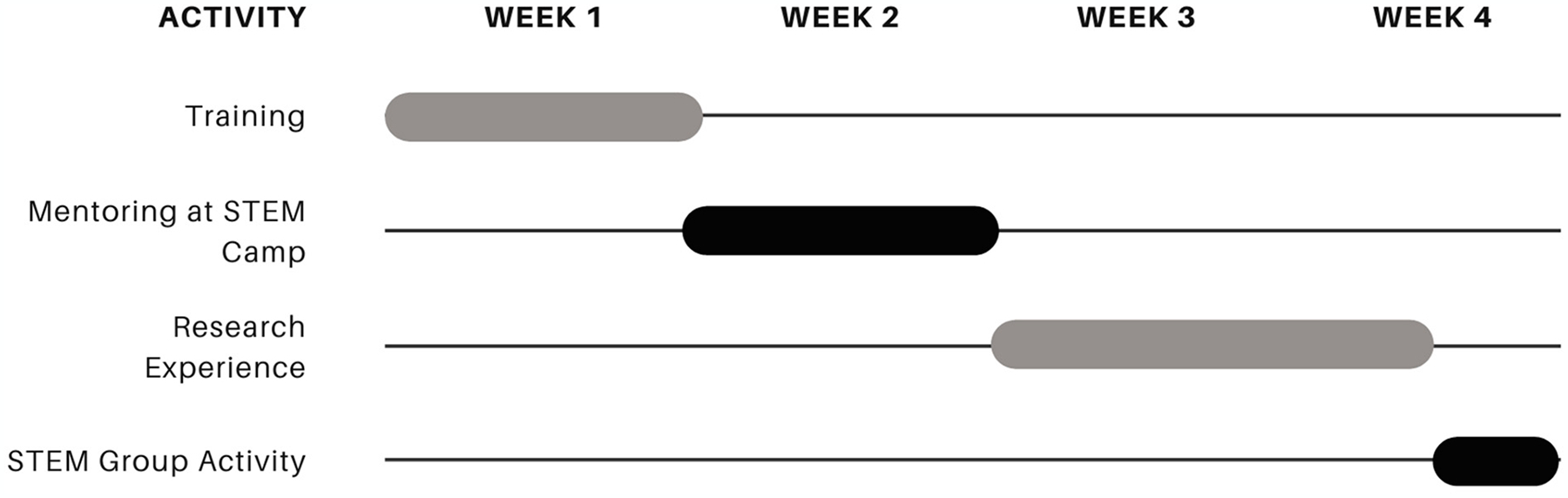
Schedule of the 4-week START summer program.

The peer mentor training is offered in partnership between the UK START Program and the department of Transformative Learning at the University of Kentucky as a part of the three-prong approach to creating a cohesive coaching culture at the institution (J. A. Bradley et al., 2024). Because high school participants in the START program act as mentors in the See Blue STEM Camp (described below) but are also mentored by undergraduate and graduate students in their assigned laboratories, they receive the same mentorship training as the postsecondary mentors, which has been accredited by the College Reading and Learning Association. Figure 1 depicts the START student program pipeline, showing the high school START Apprentices as both mentors and mentees in the program (L. H. Bradley et al., 2021). Figure 2 depicts a typical summer experience for the START Apprentices. In week one, they receive program onboarding and trainings, including peer mentor training. In week two, START Apprentices serve as near-peer mentors at the See Blue STEM summer camps for elementary and middle school students. In weeks 3 and 4, START Apprentices participate in authentic research experiences in active academic research laboratories and finish the program with a STEM-related team building activity.

During the three-hour long training, which occurs in the first week of the summer program (Figure 2), START Apprentices learn coaching-based tools and the Appreciative Coaching Framework for working with mentees (J. A. Bradley et al., 2024). The coaching tools include the three levels of listening (Cook & Poole, 2011), promoting growth mindsets (Baldwin et al., 2020), and foundational coaching question-asking (J. A. Bradley et al., 2024). As previous research has explored the differences between coaching and advising (Sepulveda & Birnbaum, 2022), the peer mentor training encourages participants not to give advice but, instead, to ask clarifying and encouraging questions so that the mentee feels empowered to create a plan of action and motivated to accomplish their goals. Additionally, mentors are trained to refrain from asking why questions, instead choosing questions that begin with how or what, to avoid making their mentee feel like they need to defend their answer. This coaching question approach has been found to benefit future mentors, as individuals do not feel the need to have all the right answers so long as they ask the right questions (McFarlane, 2023).

**Figure 3.**
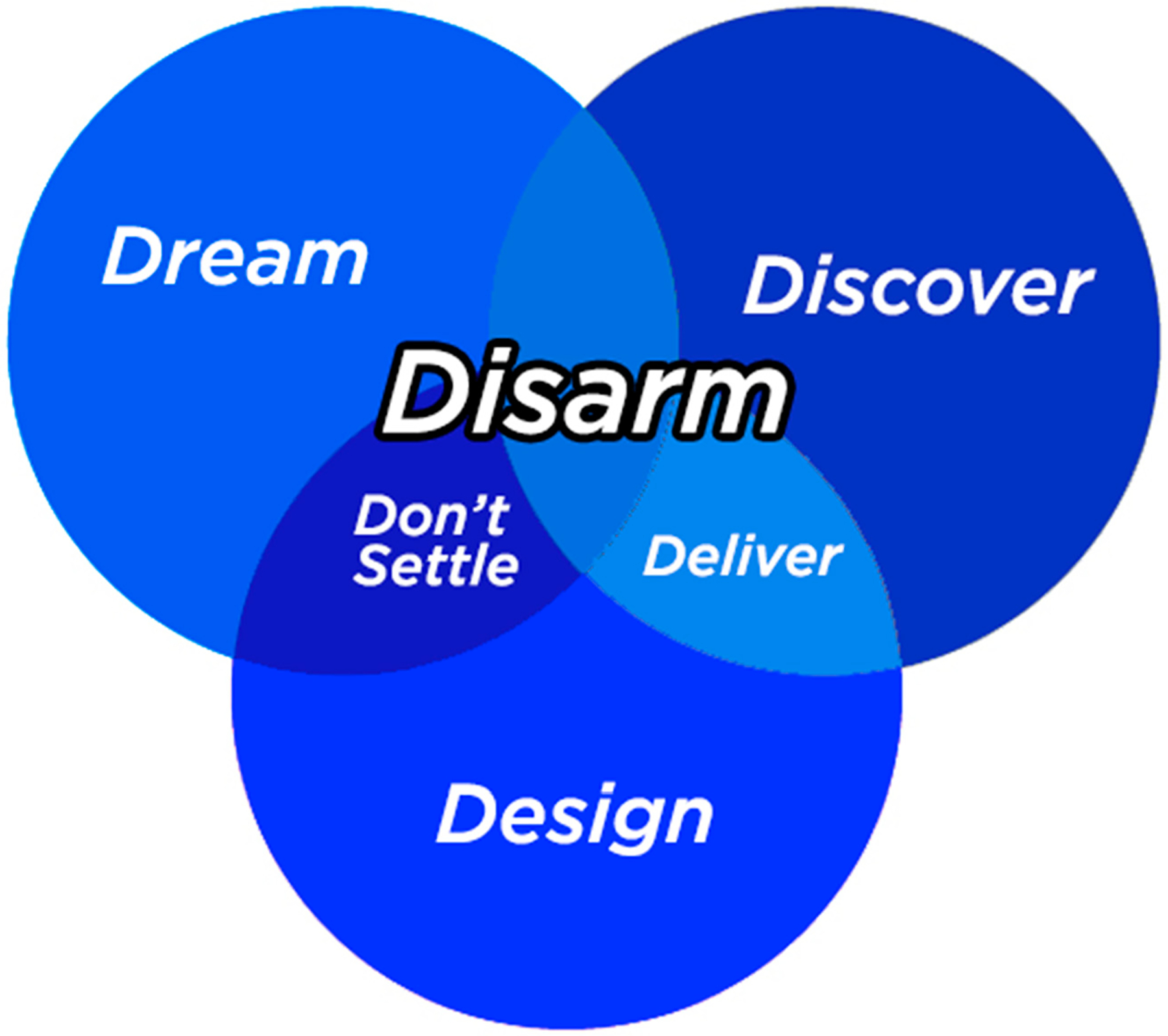
Appreciate coaching framework training emphasis.

Additionally, the training covers the Appreciative Coaching Framework (J. A. Bradley et al., 2024), which has evolved from the Appreciative Advising framework (Bloom et al., 2008). The coaching framework is meant to address the holistic success of students and consists of six phases: (a) Disarm, (b) Discover, (c) Dream, (d) Design, (e) Deliver, and (f) Don’t Settle. While near-peer mentors are not required to utilize all phases in all sessions with their mentees, students are expected to focus on the disarming phase of the coaching model, in which students create a safe and welcoming environment for the mentee as well as openly valuing the student (J. Bradley & Reynolds, 2021). In this disarming phase, “coaches ‘set the stage’, ensuring that all the environments with which the student may come in contact are welcoming.” (J. Bradley & Reynolds, 2021, p. 16). This can be done through a number of means, such as greeting the student and thanking them for attending the coaching session as well as smiling and using engaging small talk about the student’s interests and hobbies. Near-peers were encouraged to disarm their students by asking them questions to learn about each mentee as an individual, making eye contact and being friendly, as well as providing gentle encouragement to the campers to take the lead on all science experiments and activities (J. Bradley & Reynolds, 2021; J. A. Bradley et al., 2024). Figure 3 shows the peer mentor training’s emphasis on the Disarm phase of the Appreciative Coaching Framework while indicating how the other phases can often occur simultaneously. Additionally, to ensure that students are prepared to coach mentees in the moment, using both the coaching tools and the Appreciative Coaching Framework, opportunities for practice are provided through role-playing.

The UK START peer mentor training equips START Apprentices with essential mentorship and leadership skills. This training, which mirrors the mentorship training received by postsecondary mentors, ensures that the high school mentors are well prepared to support and guide their mentees effectively in a hands-on, interactive STEM learning environment, such as those found in the See Blue STEM Camp.

### 3.2. See Blue STEM Camp

The See Blue See STEM summer camp offers elementary and middle grades students an informal setting designed to specifically engage students from minoritized populations in STEM. The camp aims to harness the transformative power of hands-on, interactive sessions with STEM professionals, providing a unique and impactful learning experience (Roberts et al., 2018).

STEM activities include tasks such as building robots using Lego or Vex platforms (Mohr-Schroeder et al., 2014) and using 3-D pens to create structures (Roberts et al., 2018). These activities have been found to increase camp participants’ interests in STEM careers (Mohr-Schroeder et al., 2014) and provide more context and purpose for STEM subjects in school, like mathematics and science (Roberts et al., 2018). The START Apprentices participate in the summer learning opportunity as group co-leaders primarily coaching and scaffolding campers on completing the STEM activities, but they also help to facilitate and encourage students throughout the day while working with large groups of campers. Through this type of mentoring experience, UK START Apprentices are exposing younger students to the benefits of engaging STEM experiences while also practicing the foundational coaching skills from the mentor training.

## 4. Methods

### 4.1. Research Context

An exploratory research design was utilized to investigate the experiences and outcomes of the START Scholar cohort, consisting of 23 participants from the 2023 summer session. Of the overall Summer 2023 cohort, approximately 48% of respondents identified as female. Nearly a quarter of the START Apprentices identified as being first-generation, and 22% indicated having a long-lasting or chronic condition that substantially limited one or more of their major life activities. Additionally, 26% of the Apprentices identified as Black or African American, 8% identified as Asian, 8% identified as Hispanic or Latinx, 8% identified as more than one race, and 4% identified as Middle Eastern.

This study took place at the conclusion of the START summer session, which ran for four weeks (Figure 2), through the month of June 2023. The START peer mentor training had occurred approximately one month before the interviews, during the first week of the summer cohort, and student participants subsequently worked as mentors in the STEM camp during the second week of the program. Using an exploratory research design allowed researchers to delve into the nuances of the Apprentices’ mentorship and leadership development, as well as their interactions within the See Blue STEM Camp. By employing the qualitative interview method, the research aimed to capture a comprehensive understanding of how the START program influenced the participants’ skills and perspectives. This design was particularly suited for uncovering new insights and generating hypotheses for future studies on near-peer mentoring and STEM education. The research design was submitted and approved by the University of Kentucky’s Institutional Review Board.

### 4.2. Data Collection

Data were collected for this research via semi-structured interviews that were held with participants at the conclusion of the START program. START Apprentices in the summer cohort had the opportunity to opt into interviews and 19 START Apprentices agreed to speak with interviewers after the one-month summer programming. The interviewers virtually met one-on-one with students for 30 min to learn more about their experiences in START, including their training and work as near-peer mentors at the camp, challenges from their perspectives, the students’ takeaways after their participation, and any future interests related to their experiences. START Apprentices were additionally asked two questions about their experiences training and working as mentors: (a) what were their takeaways from the START peer mentor training and (b) how did the training impact their work with the STEM camp mentees? The audios of these interviews were recorded and later transcribed by program personnel. To answer the current study’s research question, responses were coded for themes related to their mentoring training and experiences.

### 4.3. Data Analysis

To answer the current study’s research question, interview transcripts were coded by two graduate students working on the grant using a priori and post hoc methods related to their mentoring training and experiences. The MAXQDA software (Version 20.4.2) was used to compile and organize codes. With the a priori coding, transcripts were analyzed in alignment with the interview protocol, and additional findings that were not initially anticipated by the research team were captured in the post hoc coding. The codes for the research questions were the compared across interviews to generate relevant themes in relation to student participants’ START peer mentor training and volunteering in the STEM camp.

## 5. Findings

Four main themes emerged from the coding of interviews for references to the near-peer mentoring component: Highlights of mentoring, challenges of mentoring, personal growth from mentoring, and future interests in mentoring. The personal growth section was further broken down into three general categories: leadership, awareness of STEM knowledge, and understanding of how to be a mentor.

### 5.1. Training Takeaways

Overall, START Apprentices appeared grateful for the knowledge and preparation provided by the training. For example, one student compared it to previous experiences in a similar role:

The summer before this, I worked at [mentoring program] doing camps with kids, and they did not give me any sort of training. The just told me, go in there and make sure they don’t kill each other, pretty much. And so, it was nice to actually, like, kind of, get talked to a bit on how to handle it. (…) It was good to have some prep rather than just being thrown right into it.

However, when asked about specific takeaways from the mentor training, two main themes emerged in the interviews with START Apprentice participants: improved communication with their mentees and their ability to maintain composure in stressful mentoring situations. Additionally, START Apprentices reflected on how this role as a trained mentor impacted their leadership abilities and perceptions of their own STEM knowledge.

### 5.2. Communication Skills

In identifying takeaways from the mentor training, START Apprentices primarily acknowledged their newly acquired coaching communication skills. For example, they became more aware of how their words and responses could be interpreted by their mentees, becoming more intentional about how they communicated. One Apprentice explained, “You don’t want to make them feel bad, like STEM is bad, education is bad. You know? So I found that, you know, sometimes you have to—there are ways to talk to them”. Another mentor became more aware of the effects of why questions by stating the following:

It kind of made me, I don’t know, more aware in a way. Because, like, I didn’t realize until before, but I asked a lot of ‘why’ questions before, and then I remember on the first day, I asked a lot of why questions to the kids, because it was just what I automatically thought of, and they were—they did not know what was going on. But then, when I used the skills that we learned, it helped a lot more later on.

Similarly, another focused on why questions in their response:

It kind of prepared me for like questions I could ask for them to be more engaged with me or get to know me. I felt like more comfortable. Yeah, and not saying like, ‘why’ as much because it sounds kind of judgmental for them.

In fact, judgment was a common theme, with another student saying, “It impacted me to be mindful to them, not to judge them with anything that they have, answer the questions that they have with honesty, and make sure that’s not judgmental in any way”.

Other START Apprentices focused on the reciprocal learning role of the mentor, which related to asking open-ended coaching questions. In this sense, mentors were encouraged to give mentees space to formulate their own plans for success by refraining from giving commands. In this sense, they appreciated the straightforward nature of the training. One participant said, “it made the experience more, like—easier, because we knew our do’s and don’ts”.

In discussing specific takeaways of the training, one respondent noted, “I think that asking the open-ended questions, it helped. I feel like that helped the student feel more comfortable in talking—with talking, because I understand that some of them might have been intimidated because we’re bigger or whatever”. Similarly, one START Apprentice focused on refraining from giving commands and stated, “I think it helped us because we had to phrase things very specifically. We couldn’t like give commands. We had to kind of like ease them into it”. Finally, another student said the following of the training:

My takeaway from that was that the kids are a mixed bag, right? So, I’m gonna have to just be kind, right? But I’m also—when I’m asking them things and trying to help them figure thing out, I can’t feed them the answers. I have to, like, try and get them to reach that conclusion by using, like, ‘what if’ or ‘how would you get there?’ That kind of stuff.

These findings highlight that the START Apprentices primarily gained valuable coaching communication skills. They became more intentional with their language, avoiding judgmental tones and focusing on open-ended questions to foster a supportive environment. They appreciated the straightforward nature of the training, which emphasized the importance of not giving commands but rather encouraging mentees to develop their own plans for success. This approach helped them feel more comfortable and effective in their roles, ultimately benefiting both mentors and mentees.

### 5.3. Maintaining Composure

START Apprentices also discussed an unforeseen benefit of the training: strategies for maintaining composure while working with mentees. For instance, one student said “I really took away a lot of the training how to manage my stress and emotions”. Another stated, “I think I was able to stay a little calmer, like if anyone was like crying or anything, I knew how to better approach it”. Even though the topic was not an intended focus of the mentor training, the ability to maintain composure in stressful situations while working with students in camp became an important skill.

Given the age group of the mentees and the number of children at camp with whom the mentors were working, mentorship extended beyond simply coaching around STEM knowledge and activities, often including the campers’ behavior and interactions with one another. One Apprentice noted that working with such a young age group raised their anxiety. Therefore, they needed to adapt what they had learned in the training to not only coach the students on the STEM content but also on their participation in the camp. One START Apprentice reflected on how they would recall the training in moments of high stress:

When I was in the camp, there were always kids that would push a button or annoy me, so I would always think back to the exercise, and I would just like take a few deep breaths and then go back. Or sometimes there’d be kids fighting and trying to get on each other’s nerves, and I’m just like, ‘Okay, take a deep breath’, and then figured out the problem.

UK START Apprentices identified an unexpected benefit of the mentor training: strategies for maintaining composure while working with mentees. They learned to manage stress and emotions, which proved crucial when dealing with young campers’ behavior and interactions. They adapted their training to handle not only STEM coaching but also the overall participation and conduct of the children, using techniques like deep breathing to stay calm in high-stress situations. This skill became essential for effectively managing the dynamic camp environment.

### 5.4. Leadership Skills

START Apprentices acknowledged that assuming the role of a trained mentor in the STEM camps produced internal growth as well. Many began to see themselves as either a leader or a role model to the individuals they were mentoring. One student said of their leadership growth, “I think I learned a lot. Not just about STEM, but like how to be a leader and how to care for people and communicate”. Another participant described themselves as a “role model” for both the mentees in the camp and other young people in their life who expressed an interest in STEM. Similarly, one participant saw START mentorship as the beginning of future opportunities to work in a similar capacity, “Interacting with the kids and stuff—I will probably try to do that more when looking for summer jobs and stuff, like working at camps, because I really enjoyed that”.

Finally, START Apprentices also gained greater insight into what it meant to be in a leadership role. Several students mentioned that their idea of a mentor changed after participating in the START Program on both sides of the mentoring relationship. One student said the following:

I could go up to like someone, and it’s not like school, where it’s like you go up to your teacher, and it’s like, ‘you know what’s the answer to this or what’s the answer to that?’ It’s like you’re solving something with a partner, right? Like, you’re both trying to solve a problem. It’s not like learning something like that. It’s like, - yeah, it’s like problem-solving.

UK START Apprentices reported significant internal growth from their roles as trained mentors in the STEM camps. Many began to see themselves as leaders or role models, gaining skills in leadership, communication, and caring for others. They also recognized the potential for future opportunities in similar roles, expressing interest in working at camps or in mentorship positions. Additionally, their perceptions of mentorship evolved, viewing it as a collaborative problem-solving process rather than a traditional teacher–student dynamic. This shift highlighted the reciprocal nature of the mentoring relationship, where both mentors and mentees learn and grow together.

### 5.5. Awareness of STEM Knowledge

The last theme to emerge from the interviews was how assuming the mentorship role reaffirmed participants’ confidence in their STEM knowledge. Many noted that an impact of the training was being more comfortable in helping mentees when they didn’t understand something related to the STEM tasks at camp. One mentor stated, “I felt smart with the kids. (…) They did these like robotic things, and I ironed a lot of stuff about that, so that I got to help them out, which made me feel pretty cool”. Similarly, another participant stated that the role of mentor helped to clarify not only what they were learning from the program, but also what knowledge they already brought to the role:

There was lots of little things that, sort of like, add up. Like, when I was working with the kids. You know, I didn’t know—I don’t know anything about those robots, but just like slowly figuring it all out, that was kind of neat. And like, I knew a lot of stuff already, as well as what I learned. But it was kind of cool, like, ‘no way’. Like being able to be like, ‘Oh, I knew that’ or being able to answer questions. And you know, I learned a lot. I learned a ton, but it was just cool seeing what I did know.

UK START Apprentices acknowledged that serving as trained mentors in the STEM camps fostered their internal growth. They began to see themselves as leaders and role models, gaining valuable skills in leadership, communication, and empathy. Many expressed a newfound interest in pursuing similar mentorship roles in the future, recognizing the rewarding nature of these experiences. Additionally, their understanding of mentorship evolved, viewing it as a collaborative effort where both mentors and mentees work together to solve problems, rather than a traditional teacher–student relationship. This shift highlighted the mutual benefits and growth opportunities inherent in the mentoring process.

### 5.6. Program Challenges

In addition to our findings, we gained insight into challenges of this new program. First, as mentioned in Section 5.3, some Apprentices in general found it hard to mentor older students (e.g., grades 5–8 students) at the STEM camps. One Apprentice stated that they wished there had been specific training for engaging middle school age groups, as mentors for this group had different needs than the ones working with the younger elementary students. Additionally, the messaging could have been improved as a few START Apprentices did not appreciate or expect to have a service component within the 4-week program. One participant stated, “it wasn’t exactly what I had expected from the program, especially with watching over the kids. I could have, like, misread something when it came to the program itself, but I had first assumed we would be in the labs pretty much the entire time”. Finally, the data collection piece was difficult as it was challenging to connect with UK START Apprentices after the program to have the interviews. After the program, many Apprentices likely wanted to enjoy the remainder of their summer and so the researchers needed to follow up frequently to schedule and complete the interviews.

## 6. Discussion and Conclusions

### 6.1. Discussion of Study and Overall Themes

The current study examined the effects of a coaching-based approach to peer mentor training through START Apprentices’ interviews at the conclusion of the UK START program. The analysis of interview data from the START Apprentice program revealed several significant themes regarding the near-peer mentoring component. Four primary themes emerged: highlights of mentoring, challenges of mentoring, personal growth from mentoring, and future interests in mentoring. Within the personal growth category, three distinct areas were identified: leadership development, STEM knowledge awareness, and mentorship understanding.

Our findings align with the established literature emphasizing the benefits of authentic learning experiences and mentorship in STEM (Edmonds-Cady & Sosulski, 2012; Mohr-Schroeder et al., 2014; Roberts et al., 2018; L. H. Bradley et al., 2021). The training provided to START Apprentices proved to be a crucial foundation for their mentoring experience. The structured training provided to START Apprentices, which they consistently appreciated compared to previous experiences lacking formal preparation, reinforces the importance of intentional mentor preparation (Lunsford et al., 2017). This aligns with the call for more studies evaluating the effectiveness of mentorship training programs, particularly those utilizing a coaching approach (Morales et al., 2020; McFarlane, 2023).

Consistent with prior research highlighting the development of communication skills and leadership qualities in mentors (Tenenbaum et al., 2014; Kalpazidou Schmidt & Faber, 2016), our study found that apprentices reported significant improvements in their coaching abilities. They developed a greater awareness of their language’s impact, learned to use open-ended questions, and fostered a supportive environment for mentees. This supports the idea that mentors learn valuable professional skills through these experiences.

However, our study advances beyond existing literature in several key areas. First, we identified an unexpected but crucial outcome: the development of emotional regulation skills. Apprentices reported that the training helped them manage stress and maintain composure while working with young campers, particularly in high-stress situations. This skill proved essential when dealing with behavioral challenges and interpersonal conflicts among mentees, with many apprentices implementing specific strategies like deep breathing to maintain their effectiveness as mentors. This finding highlights a unique benefit of coaching-based mentor training that has been less emphasized in prior research. The ability to regulate emotions is a vital skill for STEM professionals, and our study shows that near-peer mentoring can serve as a conduit for developing this competency.

Second, our research demonstrates the profound impact of near-peer mentoring on STEM knowledge self-efficacy. While previous studies have noted that mentors gain content knowledge (Tenenbaum et al., 2014), our findings show that the act of guiding younger students significantly strengthens mentors’ confidence in their STEM abilities. Through the process of guiding younger students, apprentices gained clarity about their existing knowledge while continuing to learn. This dual process of teaching and learning, resulting in enhanced subject matter expertise and self-assurance, highlights the reciprocal benefits of peer mentorship (Colvin & Ashman, 2010), or in this context, near-peer mentorship. This finding is particularly significant for underrepresented populations, as increasing STEM self-efficacy is crucial for fostering positive STEM identities and success (Ford et al., 2023; Singer et al., 2020).

Further, our study provides empirical evidence for the effectiveness of a focused coaching training in preparing near-peer mentors. The training proved to be a critical foundation, enabling apprentices to effectively apply coaching principles in their mentoring roles. This demonstrates that even short training sessions can yield significant benefits, providing valuable insights for designing efficient and impactful mentor training programs in higher education.

Finally, while Luedke et al. (2023) highlighted the importance of mentors who “looked like” their mentees, our study further shows the specific mechanisms through which mentors benefit, regardless of shared identities. The focus on coaching skills, emotional regulation, and STEM self-efficacy provides a deeper understanding of the mentor’s growth, adding to the understanding of the generalized positive effect of mentorship, which is an advancement beyond the focus of shared identity.

These findings suggest that structured near-peer mentoring programs, particularly those incorporating coaching-based training, can create multi-faceted benefits for mentors. These benefits extend beyond supporting younger students in STEM, serving as valuable professional development opportunities for emerging STEM practitioners. The development of communication skills, emotional regulation, leadership capabilities, and STEM confidence indicates that such programs can contribute significantly to the growth and retention of future STEM leaders.

### 6.2. Conclusions and Implications

The findings of this study showed the importance of providing training to increase mentors’ communication skills, such as open-ended question asking and active listening, in addition to building participants’ confidence in their leadership and STEM capabilities. Given that some participants expressed shock and gratitude at receiving training, comparing the experience to past mentorship opportunities without such support, supports that mentoring programs should be intentional in preparing individuals to assume these leadership opportunities.

However, it is worth noting that this sample is quite unique, given both the age of the START Apprentices (grades 11 and 12) as well as the dual experience as both mentor and mentee during the program. Because most near-peer mentoring programs in higher education employ undergraduate and graduate mentors, future research should compare training outcomes among these populations to the takeaways experienced by the high school START Apprentices. Additionally, in acting both as mentors and mentees over the course of the summer session, START Apprentices may have some bias towards the effects of foundational coaching skills after being on the receiving end of the mentorship produced by the training. Thus, further examinations of these outcomes from samples with less of an opportunity for bias should be considered by future researchers.

Despite these limitations, the findings strongly support the effectiveness of a coaching-based approach to mentor training. This approach relieves mentors of the pressure to be all-knowing experts and instead empowers them to facilitate learning through thoughtful questioning and guided discovery. This strategy appears particularly valuable for individuals from underrepresented populations in STEM fields, as it positions them as knowledge experts for younger students while building their own confidence and leadership skills.

For organizations with missions similar to the UK START program, these findings suggest two key recommendations: First, the integration of near-peer mentoring components can provide valuable development opportunities for student participants. Second, the implementation of coaching-based mentor training appears crucial for maximizing the success of these mentoring relationships. These elements together create a supportive environment that benefits both mentors and mentees while advancing broader goals of STEM education and inclusion.

## Data Availability

Due to the potentially identifiable nature of the open-ended data as well as the age of the participants, the data are not publicly available.
